# Earthworm (*Pheretima aspergillum*) extract stimulates osteoblast activity and inhibits osteoclast differentiation

**DOI:** 10.1186/1472-6882-14-440

**Published:** 2014-11-11

**Authors:** Yuan-Tsung Fu, Kuo-Yu Chen, Yueh-Sheng Chen, Chun-Hsu Yao

**Affiliations:** School of Chinese Medicine, China Medical University, 91 Hsueh-Shih Road, Taichung, 40402 Taiwan; Department of Chinese Medicine, Taichung Tzu Chi Hospital, The Buddhist Tzu Chi Medical Foundation, Taichung, 40427 Taiwan; Department of Chemical and Materials Engineering, National Yunlin University of Science and Technology, Yunlin, 64002 Taiwan; Department of Biomedical Imaging and Radiological Science, China Medical University, Taichung, 40402 Taiwan; Department of Biomedical Informatics, Asia University, Taichung, 41354 Taiwan

**Keywords:** Earthworm, Osteoblasts, Osteoclasts

## Abstract

**Background:**

The potential benefits of earthworm (*Pheretima aspergillum*) for healing have received considerable attention recently. Osteoblast and osteoclast activities are very important in bone remodeling, which is crucial to repair bone injuries. This study investigated the effects of earthworm extract on bone cell activities.

**Methods:**

Osteoblast-like MG-63 cells and RAW 264.7 macrophage cells were used for identifying the cellular effects of different concentrations of earthworm extract on osteoblasts and osteoclasts, respectively. The optimal concentration of earthworm extract was determined by mitochondrial colorimetric assay, alkaline phosphatase activity, matrix calcium deposition, Western blotting and tartrate-resistant acid phosphatase activity.

**Results:**

Earthworm extract had a dose-dependent effect on bone cell activities. The most effective concentration of earthworm extract was 3 mg/ml, significantly increasing osteoblast proliferation and differentiation, matrix calcium deposition and the expression levels of alkaline phosphatase, osteopontin and osteocalcin. Conversely, 3 mg/ml earthworm extract significantly reduced the tartrate-resistant acid phosphatase activity of osteoclasts without altering cell viability.

**Conclusions:**

Earthworm extract has beneficial effects on bone cell cultures, indicating that earthworm extract is a potential agent for use in bone regeneration.

## Background

Large bone defects caused by trauma, inflammation, tumor resection, or skeletal abnormalities are a considerable challenge to reconstructive surgeons. Numerous traditional Chinese medicines are used to treat bone-related diseases, including osteoporosis, bone fracture, and arthritis. Many clinical and animal studies have demonstrated that they have therapeutic effects on bone regeneration [1, 2]. The earthworm is widely used in traditional oriental medicine to treat cardiovascular diseases and antipyretic [3, 4]. The earthworm’s body cavity contains coelomic fluid, which has several biological activities such as antibacterial, hemolytic, agglutinative, and mitogenic activities [5–9]. Increasing amounts of evidence indicate that earthworm extract has various beneficial pharmacological activities, including fibrinolytic and anticoagulative activities, hepatoprotective activity, and antioxidative activity [10–14]. Moreover, earthworm extract can enhance wound healing and peripheral nerve regeneration [15–18]. The anti-inflammatory and antioxidant activities may be attributed to the high polyphenolic content in earthworm tissue [19]. The glycolipoprotein extract (G-90) obtained from the tissue homogenate of earthworms exhibits antioxidative, fibrinolytic, and anticoagulative activities [11, 12]. G-90 contains several growth factors, including insulin-like growth factor, immunoglobulin-like growth factor, and epidermal growth factor [13]. It also participates in tissue regeneration and wound healing.

This study evaluated the biological effects of various concentrations of earthworm extract on osteoblast and osteoclast activities via *in vitro* cell culture. To the best of our knowledge, this study is the first to evaluate the effects of earthworm extract on the bone cells activities. Bone cell activities were assessed using the tetrazolium bromide colorimetric assay, alkaline phosphatase (ALP) activity assay, matrix calcium deposition, Western blotting assay and the tartrate-resistant acid phosphatase (TRAP) activity assay.

## Methods

### Preparation of earthworm extract

Dried earthworms were obtained from the Chung Song Zong Pharmaceutical Co. (Kaohsiung, Taiwan). Their identity was confirmed by experts in pharmacognosy. Earthworm extract was prepared using a method described previously [18]. Briefly, 600 g dried earthworm was added to 7.2 l deionized water and boiled at 100°C for 1 h. The residue was volatilized at room temperature. Another extract was obtained from the pretreated earthworm by boiling deionized water for two times [water-to-earthworm ratios (ml/g) at 10:1 and 8:1] for 1 h. The aqueous extracts were filtered to remove insoluble debris and concentrated at 65°C via vacuum evaporation to 2.3 g/ml (equivalent to the dry weight of earthworms) and stored at 4°C until *in vitro* assays.

### Osteoblast culture

The cellular effects of earthworm extract on osteoblasts were assessed using the human osteoblast-like cell line MG-63 (BCRC no. 60279, Food Industry Research and Development Institute, Hsinchu, Taiwan). Cells were cultured in a growth medium consisting of Dulbecco’s modified Eagle’s medium (DMEM; Gibco, Grand Island, NY, USA), 10% fetal bovine serum (FBS; Gibco) and 1% penicillin/streptomycin (Gibco) at 37°C under 5% CO_2_ in humidified air. The medium was changed every 2 days. Adherent cells were allowed to reach roughly 80% confluence. The cells were then passaged in the culture. Passage 2 culture was used for all *in vitro* assays. Cultured MG-63 cells were seeded in growth medium in 96-well plates at a density of 1 × 10^4^ cells/well. After 1 day of incubation, the culture medium was replaced with mixed solutions of a new culture medium and various concentrations of earthworm extract at a ratio of 9:1 (v/v) [20]. In the control group, the culture medium for cell cultures was mixed with phosphate-buffered saline (PBS) at a ratio of 9:1 (v/v). After culturing for 2 days, osteoblast proliferation and differentiation were assessed by 3-(4,5-dimethylthiazol-2-yl)-2,5-diphenyl tetrazoliumbromide (MTT; USB, Amersham Life Science, Cleveland, OH, USA) assay and ALP activity assay, respectively.

### RAW 264.7 culture

The effects of earthworm extract on osteoclast proliferation and differentiation were evaluated using murine monocyte/macrophage RAW 264.7 cells (BCRC No. 60001, Food Industry Research and Development Institute). 2 × 10^3^ cells/well RAW 264.7 cells were seeded in individual wells of a 48-well tissue culture plate in a growth medium. After 1 day of incubation, osteoclast differentiation from RAW 264.7 cells was induced with 50 ng/ml RANKL (Enzo Life Sciences, Lausen, Switzerland) in α-minimal essential medium (α-MEM; Gibco) with 10% FBS for 6 days because the formation of mature osteoclasts requires 5–6 days [21–23]. The culture medium was refreshed every 2 days. After 6 days culturing, the culture medium was replaced with mixed solutions of new culture medium and various concentrations of earthworm extract at a ratio of 9:1 (v/v). In the control group, the culture medium was mixed with PBS at a ratio of 9:1(v/v) for cell cultures. After 2 days of culture, osteoclast proliferation and differentiation were determined by MTT assay and TRAP activity assay, respectively.

### MTT assay for cell proliferation

After incubation for 2 days, the supernatant was removed. Then, 10 μl MTT solution (5 mg/ml) and 100 μl culture medium were added to each well. The plate was incubated at 37°C for 4 h to facilitate the formation of insoluble formazan crystals. The solution was then removed and 100 μl/well acidic isopropyl alcohol (0.04 M HCl in isopropyl alcohol) was added to dissolve the dark-blue formazan crystals. After shaking for a few minutes, the concentration of intracellular formazan crystals was determined at a test wavelength of 570 nm against a reference wavelength of 650 nm by an enzyme-linked immunosorbent assay (ELISA) reader (uQuant; Bio-Tek Instruments, Inc., Sunnyvale, CA, USA). The number of viable cells in each well was calculated by transforming the optical density values of the MTT assay into numbers of cells/well, based on a standard curve. All experiments were performed in triplicate.

### Liu’s stain for morphological observation and osteoblast proliferation

Liu’s stain was applied to observe osteoblast morphology and proliferation [24]. After culturing for 2 days, the cell layers were rinsed three times with PBS, fixed in 2% glutaraldehyde (Acros, Geel, Belgium) for 30 min, and stained with Liu’s stain solution (30 s in solution A, followed by 90 s in solution B) (Chin Pao Co., Ltd., Taipei, Taiwan). After washing three times with deionized water to remove the remaining stain, the cell layers were observed using an inverted optical microscope (Axiovert 25; Carl Zeiss, Inc., Goettingen, Germany).

### Analysis of alkaline phosphatase for osteoblast differentiation

After 2 days of culturing, the medium was replaced with 20 μl/well 0.1% Triton X-100 (Sigma) and incubated at room temperature for 5 min for cell lysis. 100 μl/well of the commercially available ALP assay kit (Procedure No. DG1245-K; Sigma) was then added within 1 min. Absorbance at 405 nm caused by *p*-nitrophenol production was assessed for 30 min at room temperature. The change in rate of absorbance was directly proportional to ALP activity. Each experimental condition was repeated three times.

### Analysis of matrix calcium deposition

Von Kossa stain was utilized to examine the formation of the mineralized matrix [25]. Briefly, 5 × 10^4^ cells/well cultured MG-63 cells were added to the osteogenic medium (growth medium supplemented with 50 μg/ml L-ascorbic acid (Sigma), 10 mM β-glycerol phosphate (Sigma) and 10 nM dexamethasone (Sigma) in a 35-mm culture dish. The medium was mixed with various concentrations of earthworm extract at a ratio of 9:1 (v/v). In the control group, the medium was mixed with PBS at a ratio of 9:1 (v/v) for cell cultures. The medium was changed every 3 days. After culturing for 14 days, cultures were washed with PBS twice and fixed in 2% glutaraldehyde for 20 min. After incubation for 30 min with 5% silver nitrate (Union Chemical Works, Ltd., Hsinchu, Taiwan) in darkness at room temperature, cells were rinsed twice in deionized water. After drying in air, cells were exposed to ultraviolet light for 1 h until color development was complete. The cells were then immersed in 5% sodium thiosulfate (Union Chemical Works, Ltd.) for 2 min. Finally, calcification was visualized by counterstaining cells with 0.1% nuclear fast red (Sigma) dissolved in 5% aluminum sulfate (JT Baker, Phillipsburg, NJ, USA) for 5 min. Following rinsing in deionized water, matrix calcium deposition was observed with an optical microscope.

### Western blot analysis

Western blot analysis was performed to study the expression of ALP, osteopontin and osteocalcin of osteoblasts while interacting with earthworm extract. Briefly, 4 × 10^5^ cells/well cultured MG-63 cells were seeded to osteogenic medium in a 6-well culture plate. After culturing for 1 day, the culture medium was replaced with mixed solutions of a new culture medium and various concentrations of earthworm extract at a ratio of 9:1 (v/v). In the control group, the culture medium was mixed with PBS at a ratio of 9:1 (v/v) for cell cultures. The medium was refreshed every 3 days. After culturing for 7 days, adherent cells were washed twice with PBS and lysed in ice-cold lysate buffer. Cellular lysates were centrifuged at 12000 g for 30 min at 4°C. The supernatant was recovered, and the protein concentration was determined by the Bradford protein assay (Bio-Rad, Hercules, CA). 30 μg/μl protein was separated by sodium dodecyl sulfate polyacrylamide gel electrophoresis (SDS-PAGE) and transferred to the poly(vinylidene difluoride) membranes. Nonspecific binding sites were blocked with 3% skim milk in PBS for 30 min. Membranes were then incubated with primary antibodies at 1:1000 dilutions in mouse anti-ALP, osteopontin or osteocalcin for 2 days. The membranes were washed to remove unbound antibodies and then incubated with the secondary antibody (horseradish peroxidase-conjugated anti-rabbit IgG) diluted at 1:1000 for 90 min. Finally, blots were visualized by enhanced chemiluminescence (ECL) kit (Pierce, Rockford, IL, USA) using X-ray film (Konica Minolta, Japan).

### Analysis of TRAP for osteoclast differentiation

After 2 days of culturing, TRAP activity was assessed by measuring the amount of acid phosphatase (ACP) released from cells into the medium using a commercially available kit (Procedure No. 435, Sigma). Briefly, 30 μl culture media was mixed with 100 μl acid phosphatase reagent. Absorbance at 405 nm caused by *p*-nitrophenol production was observed for 30 min at room temperature. The change in rate of absorbance was directly proportional to TRAP activity. Each experimental condition was repeated three times.

### Statistical analysis

Numerical data are presented as mean ± SD. Statistical analysis was performed using one-way analysis of variance (ANOVA), followed by *post hoc* Fisher’s LSD multiple comparison test. Statistical significance was *p* < 0.05.

## Results

### Earthworm extract enhances osteoblast proliferation

**Figure 1:**
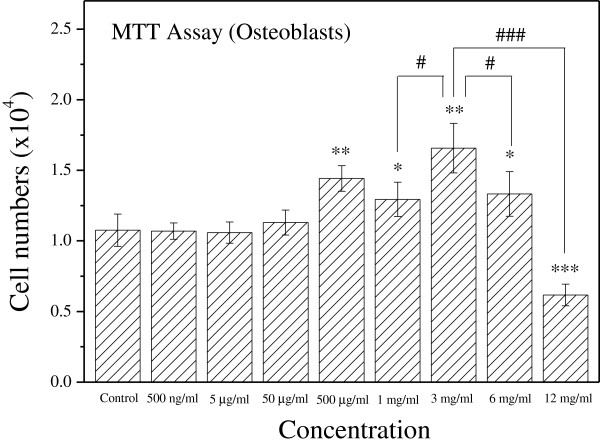
**Earthworm extract enhances the proliferation of osteoblasts.** MG-63 cells were cultured with PBS as a control or different concentrations of earthworm extract. Osteoblast viability was measured by MTT assay after 2 day of culture. (*p < 0.05, **p < 0.01 and ***p < 0.001 vs. control; ^#^ *p* < 0.05 and ^###^ *p* < 0.001 vs. 3 mg/ml).

Figure 1 shows the effects of different concentrations of earthworm extract on osteoblast proliferation measured by MTT assay after 2 day of culture. Osteoblast proliferation was dose-dependent for high concentrations of earthworm extract. Treatment with earthworm extract at concentrations from 500 μg/ml to 6 mg/ml significantly enhanced osteoblast proliferation (*p* < 0.05). Particularly, 3 mg/ml earthworm extract increased cell proliferation most (*p* < 0.01), in which the number of osteoblasts was 54% higher than that in the control group. However, the number of osteoblasts decreased significantly when the earthworm extract concentration was > 6 mg/ml (*p* < 0.05). Notably, 12 mg/ml earthworm extract significantly inhibited osteoblast proliferation (*p* < 0.001), in which the number of osteoblasts was 43% lower than that in the control group. It indicated that 12 mg/ml earthworm extract was likely toxic to osteoblasts. These analytical results were further confirmed by staining results with Liu’s stain.

**Figure 2:**
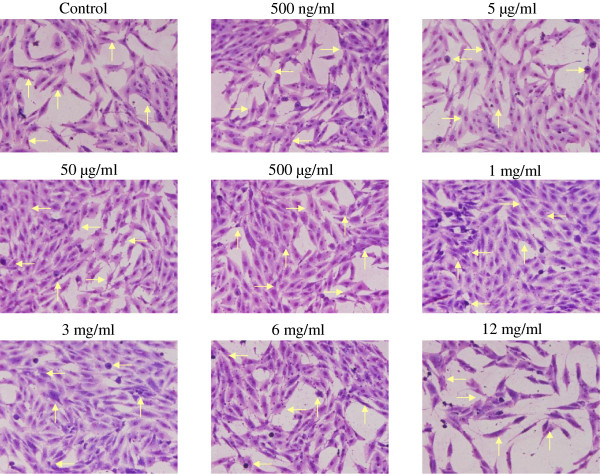
**The morphology and proliferation of osteoblasts treated with various concentrations of earthworm extract.** MG-63 cells were treated with PBS as a control or different concentrations of earthworm extract. After 2 day of culture, the cell layers were stained with Liu’s stain solution and observed using an inverted optical microscope. Arrows point to osteoblasts.

Figure 2 shows the morphology of osteoblasts cultured with various concentrations of earthworm extract for 2 days. The osteoblasts displayed spindle-shaped. The morphology of osteoblasts did not obviously differ between the control group and experimental groups. However, the number of osteoblasts increased as the concentration of earthworm extract increased from 0.5 μg/ml to 3 mg/ml, and then decreased as the concentration was raised further. Particularly, treatment with 12 mg/ml earthworm extract reduced the osteoblast population as compared with that of the control group. Similar findings were obtained by MTT analysis.

### Earthworm extract stimulates osteoblast differentiation

**Figure 3:**
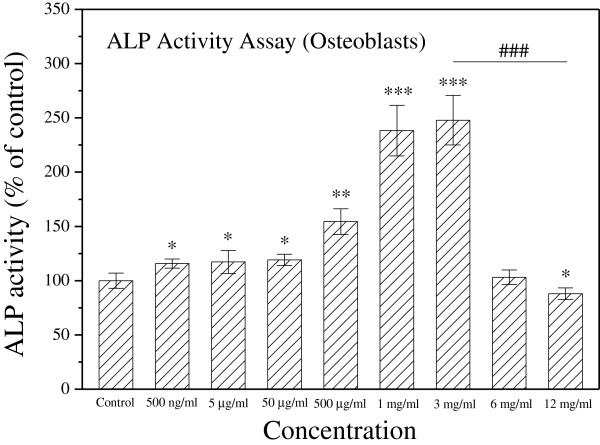
**Earthworm induces the differentiation of osteoblasts.** MG-63 cells were cultured with PBS as a control or different concentrations of earthworm extract. After culturing for 2 days, osteoblast differentiation was assessed by ALP activity assay. Results are expressed as percentage of control (*p < 0.05, **p < 0.01 and ***p < 0.001 vs. control; ^###^ *p* < 0.001 vs*.* 3 mg/ml).

ALP, a membrane-bound enzyme, is a differentiation marker of early osteoblasts. Differentiated osteoblasts exhibit elevated ALP activity, which correlates with high levels of enzyme expression [26]. Treatment of MG-63 cells with earthworm extract for 2 days stimulated the ALP activity of osteoblasts in a dose-dependent manner (Figure 3). Compared with the control group, the ALP activity was significantly increased when the concentration of earthworm extract was between 500 ng/ml and 3 mg/ml (*p* < 0.05). Notably, at higher concentrations (1 and 3 mg/ml), ALP activity increased significantly by about 1.4-fold (*p* < 0.001). However, at 6 mg/ml, ALP activity of osteoblasts did not differ significantly from that of the control group (*p* > 0.05). Moreover, at 12 mg/ml, ALP activity was significantly lower than that of the control group (*p* < 0.05).

### Earthworm extract induces matrix calcium deposition

**Figure 4:**
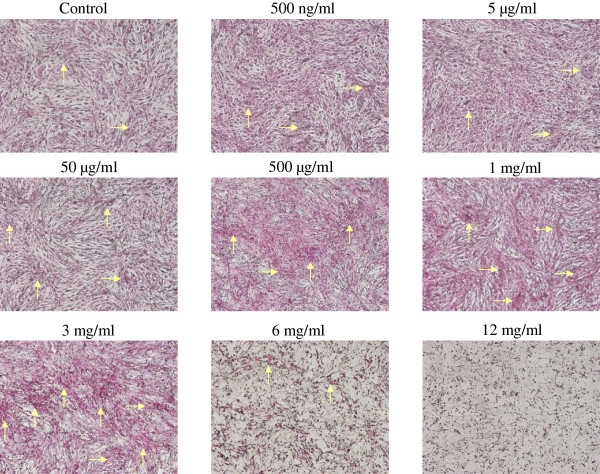
**Earthworm extract stimulates matrix calcium deposition by osteoblasts.** MG-63 cells were cultured in osteogenic medium mixed with PBS as a control or different concentrations of earthworm extract. After 14 day of culture, matrix calcium deposition was determined using von Kossa staining. Arrows demonstrate deposition of mineralized matrix.

Von Kossa staining is specific for the calcified extracellular matrix [27]. Dark staining of nodules demonstrates deposition of the mineralized matrix of bone formation. Figure 4 shows the effect of various concentrations of earthworm extract on matrix calcium deposition after 14 days of incubation. Osteoblasts cultured in medium containing 500 μg/ml to 3 mg/ml earthworm extract had high matrix mineralization. However, mineralization of osteoblasts treated with 12 mg/ml earthworm extract was not observed after culturing for 14 days.

### Earthworm extract stimulates the expression of osteogenic-related proteins

**Figure 5:**
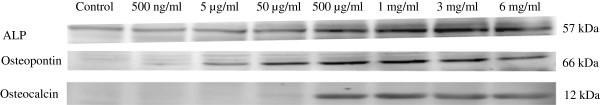
**Earthworm extract stimulates the expression of osteogenic-related proteins.** Western blot analysis was applied to observe the expression of ALP, osteopontin and osteocalcin of osteoblasts after treatment with PBS as a control or different concentrations of earthworm extract for 7 days.

Western blot analysis was applied to observe the expression of osteogenic-related proteins after treatment with different concentrations of earthworm extract for 7 days. All ALP, osteopontin and osteocalcin expression levels on earthworm extract-treated osteoblasts were higher than those of the control group (Figure 5). Cells cultured with medium containing 500 μg/ml to 6 mg/ml, 5 μg/ml to 6 mg/ml and 500 μg/ml to 6 mg/ml earthworm extract had higher upregulation of ALP, osteopontin and osteocalcin expression, respectively. Particularly, osteoblasts treated with 3 mg/ml earthworm extract had the highest ALP expression.

### Earthworm extract has no effect on the proliferation of osteoclasts

**Figure 6:**
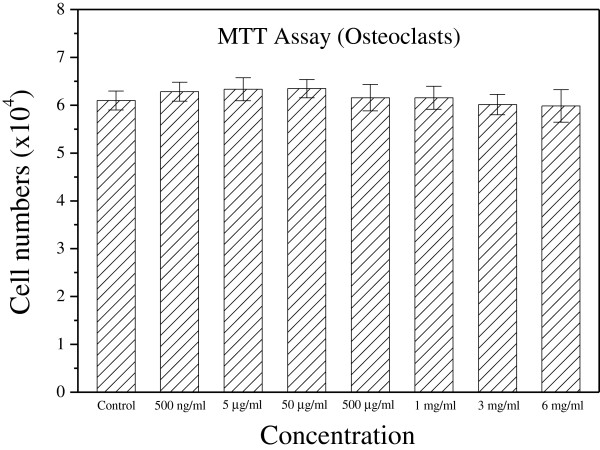
**Earthworm extract does not affect the proliferation of osteoclasts.** RAW 264.7 cells were seeded in 48-well plates and allowed to adhere for 1 day. Osteoclast differentiation from RAW 264.7 cells was induced with 50 ng/ml RANKL in α-MEM for 6 days. Osteoclasts were then cultured with PBS as a control or different concentrations of earthworm extract for 2 days. Osteoclast viability was measured by MTT assay.

The RAW 264.7 cells were used to investigate the osteoclastogenic effect of earthworm extract. Figure 6 displays the effect of earthworm extract on the proliferation of osteoclasts assessed by MTT assay after treatment with various concentrations of earthworm extract for 2 days. No significant difference in number of osteoclasts existed between the control group and experimental groups (*p* > 0.05), indicating that all test dosages (500 ng/ml to 6 mg/ml) of earthworm extract had no cytotoxic effects on osteoclasts.

### Earthworm extract inhibits the differentiation of osteoclasts

**Figure 7:**
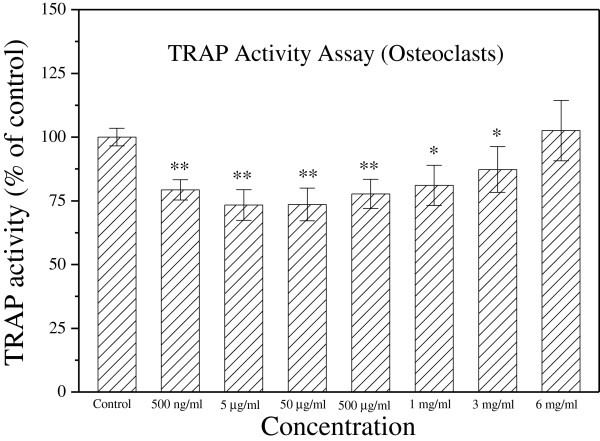
**Earthworm extract inhibits the differentiation of osteoclasts.** RAW 264.7 cells were seeded in 48-well plates and allowed to adhere for 1 day. Osteoclast differentiation from RAW 264.7 cells was induced with 50 ng/ml RANKL in α-MEM for 6 days. Osteoclasts were then cultured with PBS as a control or different concentrations of earthworm extract for 2 days. Osteoclast differentiation was determined by TRAP activity assay. Results are expressed as percentage of control (*p < 0.05 and **p < 0.01 vs. control).

The effect of earthworm extract on osteoclast differentiation was evaluated by TRAP, a marker of osteoclast differentiation [28]. Figure 7 shows TRAP activity assay results for osteoclasts cultured with various concentrations of earthworm extract for 2 days. A dose-dependent response for TRAP activity existed. Earthworm extract at concentrations < 3 mg/ml markedly decreased the TRAP activity of osteoclasts as compared with that of the control group (*p* < 0.05). Inhibition of TRAP activity was stronger when the earthworm extract concentration was < 500 μg/ml (*p* < 0.05). However, 6 mg/ml earthworm extract had no significant effect on TRAP activity (*p* > 0.05).

## Discussion

Bone remodeling, an important process in the renewal and repair of damaged bone, is initiated by osteoclastic bone resorption and subsequent osteoblastic bone formation. Previous studies have shown that several Chinese medicines significantly affect bone cell activities and enhance bone regeneration [29–33]. The earthworm has been used as a crude drug for thousands of years in China and the wider Asian region [10]. Earthworm extract can promote the differentiation of neurite-bearing cells and the proliferation and migration ability of RSC96 Schwann cells [18, 34, 35]. Chen et al. [18] found that earthworm extract markedly enhanced the nerve growth factor-mediated neurite outgrowth from PC 12 cells. Chang et al. [35] reported that treatment with 125 μg/ml earthworm extract for 24 h induced RSC96 cells proliferation. However, toxicity occurred at high concentrations (250–1000 μg/ml). Moreover, Grdiša et al. [12] found that glycolipoprotein extract (G-90) from the earthworm had a protective effect against H_2_O_2_ toxicity and stimulated the growth of human fibroblasts and epithelial cells. Additionally, Grdiša et al. [15] found that earthworm extract stimulated the synthesis of epidermal growth factor and fibroblast growth factor during wound healing on mice skin. Additionally, lumbrokinase is an important protease derived from earthworms [36]. Recent investigations have found that lumbrokinase has protective effects on hippocampus apoptosis, therapeutic potential in diabetic nephropathy and anti-ischemic action in brain [37–39].

This study evaluated for the first time the effects of earthworm extract on bone cell activities. Earthworm extract significantly promoted the proliferation, differentiation and matrix calcium deposition of osteoblasts. The most effective concentration of earthworm extract in enhancing osteoblasts proliferation and differentiation was 3 mg/ml. However, they were inhibited by high concentration (12 mg/ml), indicating that excessive earthworm extract can have an adverse effect on osteoblasts.

Osteoblasts produce ALP, osteopontin and osteocalcin, which are bone-specific markers [40]. Among these proteins, ALP and osteopontin are early markers of osteoblastic differentiation. Osteocalcin directly participates in the mineralization process and is a marker for mature osteoblasts that is expressed in the late stages of osteogenic differentiation. Moreover, osteopontin and osteocalcin are extracellular matrix proteins and crucial for bone tissue formation. The Western blot analysis results show that 3 mg/ml earthworm extract-treated osteoblasts expressed the highest level of ALP with higher levels of osteopontin and osteocalcin. It indicates that earthworm extract stimulated early differentiation of osteoblasts and is favorable for osteoblast mineralization. Calcium deposition results by von Kossa staining were in agreement with these analytical results. The most effective concentration of earthworm extract for matrix calcium deposition was between 500 μg/ml and 3 mg/ml. These results reveal that the capacity to form matrix mineralization was positively correlated with osteoblast proliferation and differentiation.

The experiments also show that 3 mg/ml earthworm extract did not significantly affect osteoclast proliferation. However, 3 mg/ml earthworm extract reduced the amount of TRAP, indicating that it inhibited osteoclast activity. These analytical results demonstrate that 3 mg/ml earthworm extract can be used for bone repair because it can promote osteoblast proliferation and differentiation as well as inhibit osteoclast activity.

## Conclusions

Our findings show that an appropriate dose of earthworm extract has potential effects on bone cell cultures. Therefore, earthworm extract might be a promising agent for bone tissue regeneration. Nevertheless, this research is only preliminary in evaluating the effects of earthworm extract on bone cell activities. Further studies are needed to identify the specific interactions between different earthworm components and bone cells and to determine the exact bioactive compounds in earthworm extract that are responsible for bone cell activities.
